# Cold Atmospheric Plasma Treatment of Chondrosarcoma Cells Affects Proliferation and Cell Membrane Permeability

**DOI:** 10.3390/ijms21072291

**Published:** 2020-03-26

**Authors:** Lyubomir Haralambiev, Andreas Nitsch, Josephine M. Jacoby, Silas Strakeljahn, Sander Bekeschus, Alexander Mustea, Axel Ekkernkamp, Matthias B. Stope

**Affiliations:** 1 Department of Trauma, Reconstructive Surgery and Rehabilitation Medicine, University Medicine Greifswald, Ferdinand-Sauerbruch-Straße, 17475 Greifswald, Germany; an124100@uni-greifswald.de (A.N.); josephinemarie.jacoby@stud.uni-greifswald.de (J.M.J.); ss166320@uni-greifswald.de (S.S.); ekkernkamp@ukb.de (A.E.); 2 Department of Trauma and Orthopaedic Surgery, BG Klinikum Unfallkrankenhaus Berlin gGmbH, Warener Straße 7, 12683 Berlin, Germany; 3 ZIK plasmatis, Leibniz Institute for Plasma Science and Technology (INP Greifswald), Felix-Hausdorff-Straße 2, 17489 Greifswald, Germany; sander.bekeschus@inp-greifswald.de; 4 Department of Gynecology and Gynecological Oncology, University Hospital Bonn, Venusberg-Campus 1, 53127 Bonn, Germany; alexander.mustea@ukbonn.de (A.M.); matthias.stope@ukbonn.de (M.B.S.)

**Keywords:** chondrosarcoma, cold atmospheric plasma, membrane functionality, growth inhibition

## Abstract

Chondrosarcoma is the second most common malign bone tumor in adults. Surgical resection of the tumor is recommended because of its resistance to clinical treatment such as chemotherapy and radiation therapy. Thus, the prognosis for patients mainly depends on sufficient surgical resection. Due to this, research on alternative therapies is needed. Cold atmospheric plasma (CAP) is an ionized gas that contains various reactive species. Previous studies have shown an anti-oncogenic potential of CAP on different cancer cell types. The current study examined the effects of treatment with CAP on two chondrosarcoma cell lines (CAL-78, SW1353). Through proliferation assay, the cell growth after CAP-treatment was determined. A strong antiproliferative effect for both cell lines was detected. By fluorescein diacetate (FDA) assay and ATP release assay, alterations in the cell membrane and associated translocation of low molecular weight particles through the cytoplasmic membrane were observed. In supernatant, the non-membrane-permeable FDA and endogenously synthesized ATP detected suggest an increased membrane permeability after CAP treatment. Similar results were shown by the dextran-uptake assay. Furthermore, fluorescence microscopic G-/F-actin assay was performed. G- and F-actin were selectively dyed, and the ratio was measured. The presented results indicate CAP-induced changes in cell membrane function and possible alterations in actin-cytoskeleton, which may contribute to the antiproliferative effects of CAP.

## 1. Introduction

Chondrosarcoma (CS) is, after osteosarcoma by about 20%, the second most common malignant bone tumor in humans [1,2]. It is usually characterized by slow growth and by the formation of hyaline cartilaginous neoplastic tissue [1]. CS affects mostly adults upwards of the sixth decade with an incidence of about three new cases per mio per year [3,4,5]. A distinction is made between primary CS, which occurs independently of the pre-existing lesion, and secondary CS, which develops on the basis of a pre-existing benign cartilage tumor such as an enchondroma or osteochondroma. A further division is made: central if the CS occurs in the medullary cavity and peripheral if it originates from the surface of the bone, generally from the cartilage cap of an exostosis. It is typical for the primary CS that they are always central, whereas secondary CS can appear central or peripheral [6]. Due to its poor vascularization, slow rate of division, and hyaline cartilage matrix, which makes access to the cells extremely difficult, the CS rarely metastasizes, and thus that only about 5%–10% of all CS have high metastatic potential [1,7]. However, its phenotypic characteristics includes resistance to chemotherapy and radiation therapy [4,5]. Another reason to consider chemotherapy as a non-efficient treatment of CS is the expression of the multidrug-resistance 1 gene P-glycoprotein [8]. Thus, surgical resection remains essential for the treatment of CS. Depending on the stage and spread of the CS, different surgical techniques are used—although for high-grade CS, a wide, en-bloc excision is necessary, for low-grade CS, an extensive intralesional curettage may be sufficient [9,10]. Thus, the prognosis of CS patients mainly depends on the histological degree and sufficient surgical resection of the tumor [1]. Therefore, it is important to look for new options in the treatment of CS. Such an option can be the treatment with cold atmospheric plasma (CAP) because of its anti-oncological effect [11]. 

With CAP being a highly energized gas at body temperature and consisting of numerous biologically active species, including reactive oxygen and nitrogen species [12,13], it seems suitable for intraoperative treatment. The inhibitory effects of CAP on tumor cells from various entities have already been demonstrated in experimental studies [14,15]. Of particular interest are the CAP treatment effects on the most common primary bone malignancy—osteosarcoma [16]. In addition to the detection of growth inhibition and induction of apoptosis by CAP treatment [17], redox signaling pathways and thus also p53-dependent apoptosis are activated at the cellular level [17,18,19,20]. CAP treatment leads in osteosarcoma cells to the changes in the permeability of the cell membrane [21] and alterations of secretion patterns [22].

The influence of CAP treatment on the cytoplasmic membrane functionality of CS cells is unknown thus far and is examined in this study.

## 2. Results

**Figure 1:**
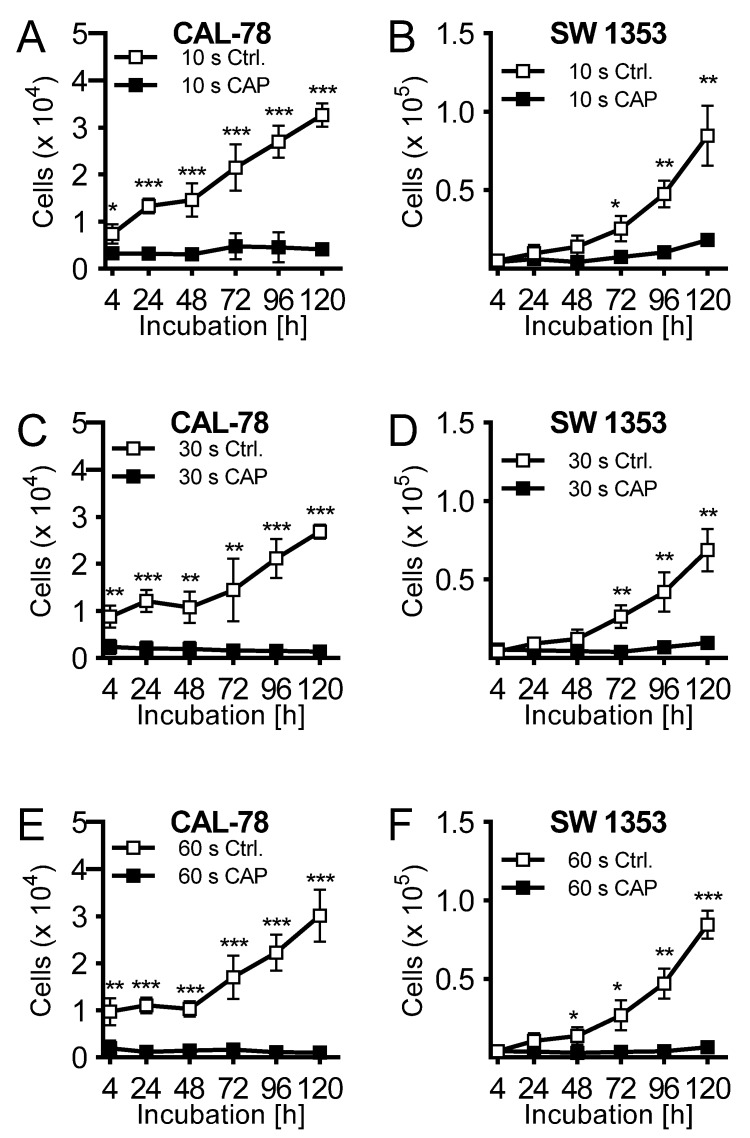
The human chondrosarcoma cell lines CAL-78 (**A**,**C**,**E**) and SW 1353 (**B**,**D**,**F**) were treated with 10 (**A** + **B**), 30 (**C** + **D**), or 60 s (**E** + **F**) cold atmospheric plasma (CAP) or carrier gas argon with kINPen med (neoplas tools, Greifswald, Germany). Living cells were counted with CASY Cell Counter and Analyzer (OLS, Bremen German) 4, 24, 48, 72, 96, and 120 h after exposure. Data are given as mean ± SD; significant differences are indicated as follows: * *p* ≤ 0.05, ** *p* ≤ 0.01, *** *p* ≤ 0.001.

The effects of CAP treatment of human cell lines of chondrosarcoma (CAL-78 and SW1353) were examined. A treatment of 10 s already demonstrated a reduction of cell growth after 120 h of incubation of 87% ± 3% (*p* = 0.043, CAL-78) and 77% ± 7% (*p* = 0.004, SW1353) compared to control cells (Figure 1A,B). The antiproliferative effect of CAP treatment increased significantly at treatment times of 30 (Figure 1C,D) and 60 s (Figure 1E,F). After the maximum treatment time of 60 s, none of the applied cell lines revealed an increase in cell numbers. Interestingly, these growth inhibitory effects were also found in the indirect treatment of the cells. Cell culture medium was treated for 10, 30, and 60 s, and immediately applied to untreated bone sarcoma cells sown 24 h before. 

**Figure 2:**
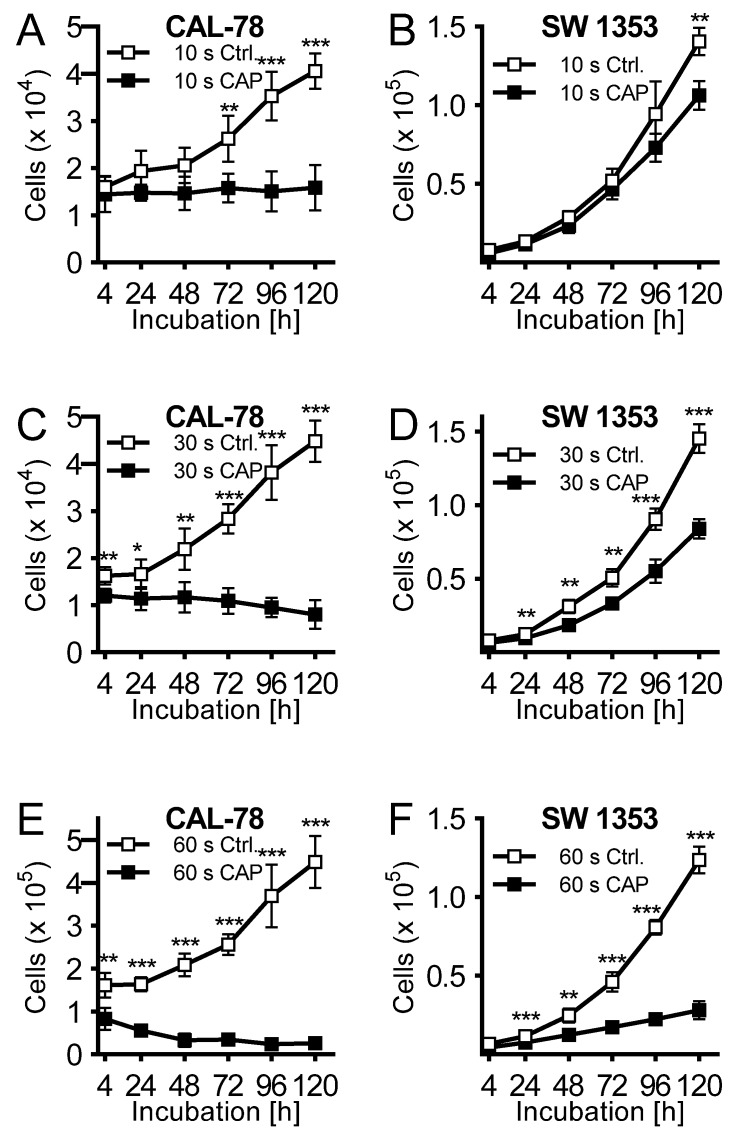
The human chondrosarcoma cell lines CAL-78 (**A**,**C**,**E**) and SW 1353 (**B**,**D**,**F**) were treated 24 h after seeding with cold atmospheric plasma (CAP). For CAP treatment, cell culture media was exposed to 10 (**A** + **B**), 30 (**C** + **D**), or 60 s (**E** + **F**) CAP or carrier gas argon with kINPen med (neoplas tools, Greifswald, Germany). Living cells were counted with CASY Cell Counter and Analyzer (OLS, Bremen, Germany) 4, 24, 48, 72, 96, and 120 h after exposure. Data were given as Mean ± SD; significant differences were indicated as follows: * *p* ≤ 0.05, ** *p* ≤ 0.01, *** *p* ≤ 0.001.

Cell culture medium was treated for 10, 30, and 60 s, and immediately applied to untreated CS cells seeded 24 h before. Cell culture medium activated with CAP for 10 s exhibited a rather modest antiproliferative effect after 120 h of incubation (CAL-78: 63 ± 7%, *p* = 0.002; SW 1353: 24 ± 6%, *p* < 0.001; Figure 2A,B). This was more pronounced when the cell culture medium was activated for 30 s (Figure 2C,D) and finally achieved a similarly strong effect after 60 s of activation as in the direct CAP treatment of the cells (Figure 2E,F).

**Figure 3:**
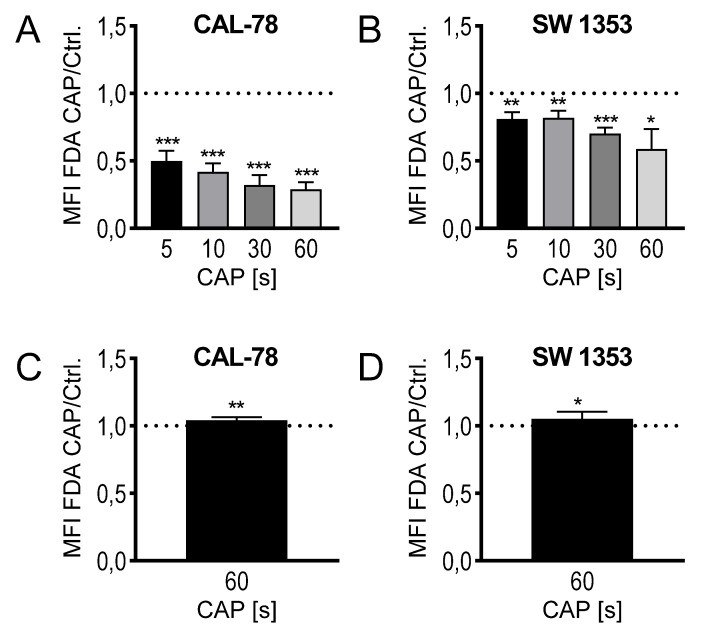
Fluorescein diacetate (FDA) uptake assay (**A**,**B**): the cell lines CAL-78 (**A**) and SW1353 (**B**) were treated with 5, 10, 30, or 60 s CAP or carrier gas argon and stained with FDA and ethidium bromide (EtBr). Afterwards, the flow cytometric analysis of single living cells was performed. Mean fluorescence intensity (MFI) of CAP-treated cells were normalized to the MFI of argon-treated cells. FDA-release assay (**C**,**D**): the cell lines CAL-78 (**C**) and SW1353 (**D**) were incubated with FDA, washed, and treated with 60 s CAP or carrier gas argon. After an incubation time of 20 min, the cell-free supernatant was analyzed in a fluorescence plate reader (excitation: 300 nm; emission 525 nm). MFI of the supernatant of CAP-treated cells normalized to the MFI of the supernatant of argon-treated cells. Data are given as mean ± SD; significant differences are indicated as follows: * *p* ≤ 0.05, ** *p* ≤ 0.01, *** *p* ≤ 0.001.

The composition of CAP as a mixture of highly reactive molecular species suggests a rapid interference with the outer cell envelope, the cytoplasmic membrane. Because biological membranes can react extremely sensitively to physical and chemical influences, the translocation of various low-molecular compounds through the cytoplasmic membrane was anlyzed. This could be considered as an indication of modifications in membrane integrity and thus membrane functionality and could therefore be part of the antiproliferative effect of CAP treatment. CS cells were treated with CAP for 5, 10, 30, and 60 s, and then incubated with fluorescein diacetate (FDA). The activity of intracellular esterases converts FDA to non-membrane-permeable fluorescein, which accumulates in the cell. The flow cytometric analysis of the cells showed a time-dependent decrease in the intracellular fluorescein concentration. After the maximum CAP treatment of 60 s, only 29% ± 5% (*p* < 0.001, CAL-78) and 59% ± 13% (*p* = 0.032, SW1353) fluorescein signal could be detected compared to control-treated cells (Figure 3A,B). The expected efflux of the nonmembrane-permeable fluorescent dye after CAP treatment was verified by a release assay. CS cells were incubated in the presence of FDA, washed, and the release of non-membrane-permeable fluorescein was detected in the cell culture supernatant. A statistically significant increase in fluorescein release can be demonstrated after CAP treatment (CAL-78: 104% ± 2%, *p* = 0.007; SW1353: 105% ± 5%, *p* = 0.026; Figure 3C,D).

**Figure 4:**
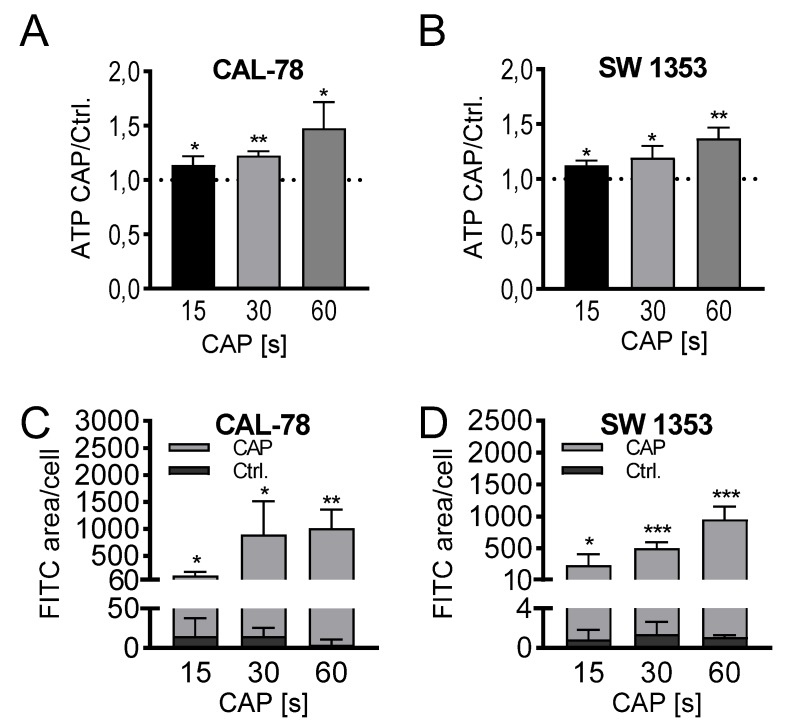
ATP release assay (**A**,**B**). CAL-78 (**A**) and SW1353 (**B**) cells were treated with 15, 30, or 60 s CAP or carrier gas argon. After an incubation period of 3 min, the relative ATP concentrations of the cell-free supernatants were measured using the CellTiter-Glo 2.0 reagent and fluorescence plate reader. The CAP treatment measurements were normalized to the control measurements. Dextran-uptake assay (**C**,**D**). CAL-78 (**C**) and SW1353 (**D**) cells were seeded on cover slides and incubated over 24 h. After incubation, cells were treated with 15, 30, and 60 s CAP or carrier gas argon. Cells were dyed with Fluorescein isothiocyanate (FITC) dextran and DAPI (4′,6-Diamidino-2-Phenylindole, Dihydrochloride). Fluorescence of FITC and DAPI were recorded with a BZ-9000 microscope and analyzed with a BZ-II Analyzer (KEYENCE, Neu-Isenburg, Germany). The FITC-positive areas were measured and normalized to the count of DAPI-labeled cell cores. Data are given as mean ± SD; significant differences are indicated as follows: * *p* ≤ 0.05, ** *p* ≤ 0.01, *** *p* ≤ 0.001.

The detection of extracellular ATP confirmed the release of low molecular weight compounds after CAP treatment. In a treatment time-dependent manner, endogenously synthesized ATP was released into the cell culture supernatant and could be detected after only 3 min of incubation. The extracellular ATP concentration increased continuously with prolonged treatment times of 15, 30, and 60 s and achieved relative concentrations of 147% ± 22% (*p* = 0.033, CAL-78) and 136% ± 8% (*p* = 0.012, SW1353) of the control preparations after 60 s of CAP treatment (Figure 4A,B).

Microscopic analyses could confirm a loss of membrane integrity. Fluorescein isothiocyanate (FITC)-conjugated 10 kDa dextran was increasingly incorporated into the sarcoma cells after CAP treatment and could be quantified fluorescence microscopically (Figure 4C,D). With prolonged treatment times, the FITC signal increased, and after 60 s of CAP treatment, the FITC signal/cell was up to several hundred times higher than in control-treated cells (CAL-78: 275-fold, *p* = 0.003; SW1353: 876-fold, *p* < 0.001). 

**Figure 5:**
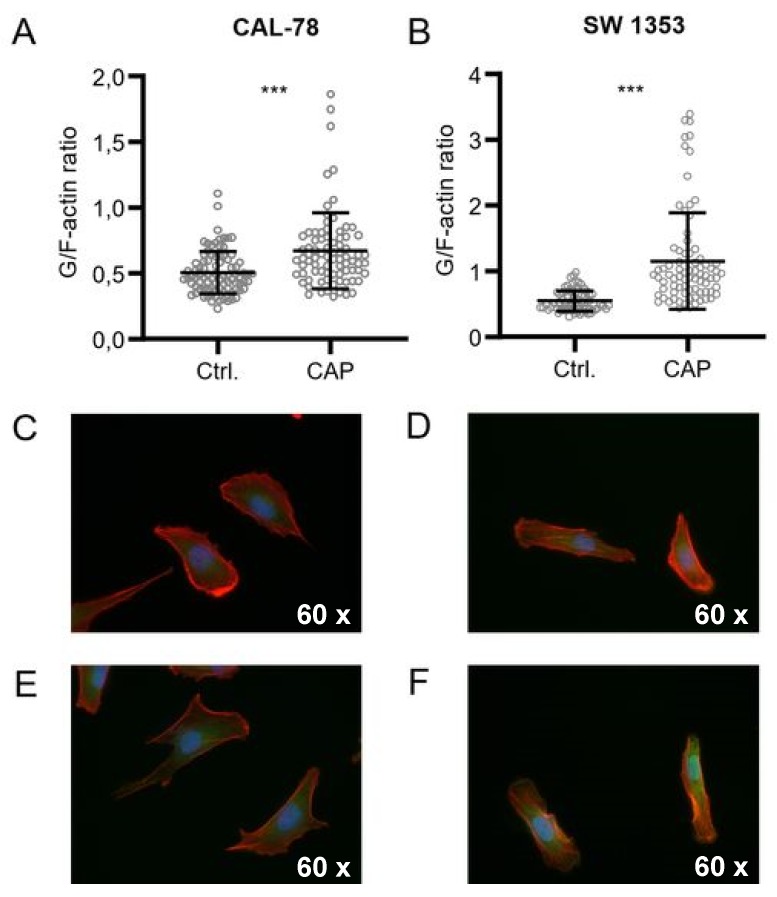
CAP treatment destroys the integrity of the cell membrane. G-/F-actin assay CAL-78 (**A**,**C**,**E**) and SW1353 (**B**,**D**,**F**) cells were seeded on cover slides and incubated over 24 h. After incubation, cells were treated with 5 (CAL-78) and 10 s (SW1353) CAP or carrier gas argon. Cells were dyed with tetramethylrhodamine (TRITC) conjugated Phalloidin and Alexa Fluor 488-conjugated deoxyribonuclease I, and fluorescence was recorded with a BZ-9000 microscope and analyzed with a BZ-II Analyzer (KEYENCE, Neu-Isenburg, Germany). The ratio of the red signal compared to the green signal of each cell was calculated. Data are given as mean ± SD; significant differences are indicated as follows: * *p* ≤ 0.05

Further microscopic analyses demonstrated that the actin composition of the cytoskeleton could be affected by CAP treatment, although the effects were inconsistent (Figure 5). The G-/F-actin ratio was significantly increased in CAL-78 (133%, *p* < 0.001) and SW1353 (209%, *p* < 0.001) cells after 5 s (CAL-78) and 10 s (SW1353) CAP exposure. 

## 3. Discussion

Although the CS is a rare osseous malignancy, its course has a high mortality rate for the affected patient. Its treatment is an interdisciplinary task in which surgical treatment plays a central role. Because of the resistance to chemotherapy and radiation therapy, further possible complementary treatment combinations of surgical tumor resection will significantly increase the chances of success of CS therapy. One such innovative procedure, which complements the intraoperative treatment of tumor tissue, could be CAP treatment of the resection margins. The antitumor effects of CAP have already been demonstrated in some cancer entities such as melanoma and pancreatic and breast carcinomas [23,24,25]. Many of the cellular effects, such as reactive oxygen species (ROS) and reactive nitrogen species (RNS) response as products of CAP treatment, have been already described in detail [26]. Possible events after CAP treatment triggered by intracellular reactive species, including DNA damage [27], lipid peroxidation [28], or mitochondrial dysfunction [29,30], have also been reported. In addition, it was shown that incubation with N-acetylcysteine (NAC,) a precursor to intracellular cysteine, counteracts apoptosis induced by CAP treatment [31]. The role of H_2_O_2_ as a by-product of CAP treatment (blood cell model) was also analyzed, with the conclusion being that it plays a dominant but not excessive role in cellular oxidation [32]. The influence of CAP treatment on the redox state of specific proteins in the cytosol was demonstrated in a prostate cell model [18]. Particularly interesting are studies carried out on the effects of CAP treatment on another primary bone sarcoma, namely, in osteosarcoma. There, CAP treatment in in vitro experiments with various osteosarcoma cell lines not only led to a reduction in cell proliferation and aktivation of apoptosis, but also to induction of transforming growth factor-beta 2 (TGF-β2), a potentially tumor suppressive factor in osteosarcoma and suppression of the angiogenic factor VEGFA (vascular endothelial growth factor A). In this way, a potentiation of these CAP effects so that it also finds a meaningful application in anti-oncological therapy is hoped for [22,33]. Yet, there are still no data available on the effects of CAP treatment on CS cells. The fact that a short CAP treatment of the CS cells leads to a long-lasting antiproliferation makes the method promising for intraoperative oncological applications. CAP treatment of only 10 seconds had dramatic effects in CAL-78 cells, with the maximum growth inhibition almost being reached. This antiproliferative effect on the other CS cell line SW1353 could only be achieved after a significant increase in the CAP treatment time (Figure 1). This individual treatment time-dependent effect of CAP exposure observed has already been described in other studies with different entities [18,21]. However, with increasing treatment time, the antiproliferative CAP effects became clear in both CS cell lines. Additionally, the CAP effects are not only limited to direct application to the tumor cells but can also be mediated by suitable liquid media such as blood or other fluids [34]. This indirect CAP treatment of CS cells led to comparable, also treatment time-dependent, but somewhat milder antiproliferative effects. Thus, indirect CAP treatment of CS cells in this study showed comparable antiproliferative effects. Although they were slightly weaker in their growth inhibition, they also depended on the treatment time. The effects of electromagnetic and thermal radiation as physical active components only occur in direct CAP treatment. Furthermore, it is likely that the CAP reactive species will react to a certain extent in the liquid solvent during indirect treatment, and thus direct CAP treatment has a significantly higher effect potential [14,35,36]. However, systemic effects can be regarded as unlikely because reactive molecular species generated by CAP are unstable in liquids [37]. The therapeutic potential of indirect CAP effects in oncology can be speculated in the inactivation of marginal infiltration with metastatic potential. The results of in vivo studies suggest that the cold plasma jet can selectively ablate bladder or melanoma cancer cells while leaving their corresponding normal cells essentially unaffected [38].

The suppressive effects of CAP treatment on proliferation and cell membrane integrity are results observed in cell culture models. There is a lack of experience with regard to the clinical use of CAP in the treatment of bone tumors in particular. Depending on the type of use, as opposed to chemotherapy, the CAP is limited to local exposure. However, because surgical tumor resection is the leading method in CS therapy, the combination with a local CAP treatment is a promising supplement in the therapeutic strategy. The great advantage of the CAP application, in contrast to other physical therapies such as thermal or laser-based procedures, is the lack of local side effects in resection tissue. As a rule, the direct physical inactivation of tumor cells leads to cytotoxic effects and necrosis, and triggers a cascade of inflammation, swelling, and pain [39]. In previous studies, apoptosis was induced in CAP-treated tumor cells regardless of the CAP source used and the tumor entity [39]. This CAP-induced controlled switching off of cells leads to the reduction of local proliferation effects in the affected tissue and ensures improved clinical tolerance of the CAP. This is certainly due to the high amounts of CAP-generated reactive species that can trigger cell cycle arrest and programmed cell death [40,41]. An important effect of CAP treatment is the changes in membrane and cytoskeletal architecture. In this way, the CAP induces a considerable malfunction of the cell and can thus also lead to its inactivation [42]. These effects have already been extensively investigated on bacterial membranes [43,44,45,46], but the effect of CAP on the eukaryotic cytoplasmic membrane has not yet been described. In this study, numerous different measurement methods (fluorescence readers, flow cytometers) were used to investigate the membrane permeability of CAP-treated CS cells. The transfer of molecules with different biochemical properties and size (FDA, ATP, 10 kDa dextran-FITC) was demonstrated. This could be shown in both directions through the cytoplasmic membrane, which means both the release from the cell and the passage into the cell.

The CAP-induced loss of ATP influences cell metabolism, which explains the antiproliferative effect of CAP treatment. An established method for the detection of membrane defects in both non-tumor and tumor cells is ATP release measurement [47,48]. In the immunological sense, extracellular ATP also serves to activate macrophages [49]. The underlying mechanisms of molecule transfer through the cytoplasmic membrane after CAP treatment are unclear, but the fact that various test molecules with different molecular weights (FDA, ATP, and 10 kDa dextran-FITC) were able to pass through the cytoplasmic membrane after CAP treatment suggests that no specific transport system was involved but rather that low molecular molecules up to a certain size are able to penetrate the cytoplasmic membrane unspecifically. Morover, the CAP properties indicate direct physical and chemical modulations of the cell membrane, such as activation of membrane-bound cellular redox mechanisms or lipid and protein oxidation [50,51].

At the present time, due to the heterogeneity of the different plasma sources, it is not generally possible to make a uniform, valid statement about CAP treatment. The CAP components lead to changes in the cytoskeleton of CS cells. The interplay of changes in the cytoplasmic membrane and in the cytoskeleton structure at the same time can have a significant influence on cell functionality and physiology. On the basis of the same mechanism of action as inactivating bacteria [51], CAP treatment of CS cells causes membrane integrity dysfunction and the loss of intracellular components as a result.

## 4. Materials and Methods

### 4.1. Cell Culture

Human CS cell lines SW1353 (American Type Culture Collection, Manassas, VA, USA) and CAL-78 (Leibniz Institute DSMZ-German Collection of Microorganisms and Cell Cultures GmbH, Braunschweig, Germany) were propagated in Dulbecco’s modified Eagle’s medium (DMEM)/F12 containing stable glutamine, 1.2 g/L NaHCO_3_, 5% fetal bovine serum, and 1% penicillin/streptomycin (SW1353) or RPMI 1640 containing 20% fetal bovine serum and 1% penicillin/streptomycin (all PAN Biotech, Aidenbach, Germany) in a humidified atmosphere at 5% CO_2_ and 37 °C.

### 4.2. CAP Device

**Figure 6:**
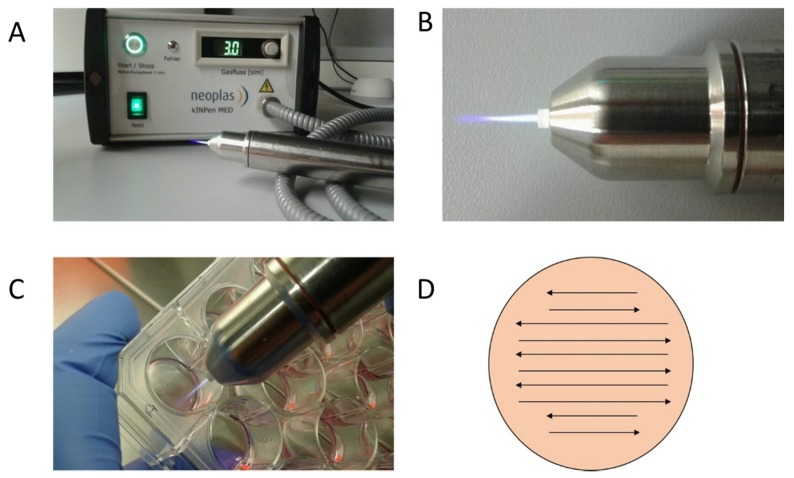
Plasma Jet kINPen MED (neoplas tools, Greifswald, Germany): detailed photos of the device in activated mode. The CAP flame is clearly visible on the CAP-Jet and therefore enables direct manual use. (**A**,**B**) Targed manual CAP treatment of the cell cultures in 24-well plates is possible thanks to the hand-friendly designed jet (**C**). In order to ensure reproducible and comprehensive CAP treatment of the cells, the CAP-Jet was moved back and forth over the plate with steady hand movements (**D**).

The CAP device kINPen MED from neoplas tools (Greifswald, Germany) was used in this work (Figure 6). This atmospheric pressure plasma jet was operated with argon as carrier gas at a flow rate of 3 standard liters per minute. It creates a 9–13 mm long plasma flame with a temperature below 40 °C. The sinusoidal operating frequency is approximately 1 MHz. The high voltage used for CAP generation is in the range of 2–3 kV. The plasma-dissipated power was specified at approximately 1 W, and the device input power at approximately 20 W. The kINPen is a CE-certified (Conformité Européenne-certification mark within the European Economic Area) product [52]. The cell treatment with the kINPen jet was performed manually. The distance was chosen so that the tip of the flame only reached the surface of the cell suspension or liquid. The flame was meandered through the liquid as shown in Figure 6B–D.

### 4.3. Proliferation Assay after CAP Exposure

Cell growth was determined after 4, 24, 48, 72, 96, and 120 h using a CASY cell counter and analyzer model TT (Roche Applied Science, Mannheim, Germany) with a 150 μm capillary. For this purpose, 1 × 10^4^ cells (SW1353) or 2 × 10^4^ cells (CAL-78) were suspended in 200 μL culture media and treated with CAP or carrier gas argon (control group) for 10, 30, and 60 s. The atmospheric plasma jet kINPen MED was used for CAP treatment. After treatment, 800 μL fresh media were added, and cells were incubated in a humidified atmosphere at 5% CO_2_ and 37 °C. Cell count was determined by suspending the cells by trypsin/EDTA (Ethylenediaminetetraacetic acid) treatment and diluting 100 μL cell suspension in 10,000 μL CASYton (Roche Applied Science). The measurement was performed three times with 400 μL each of this dilution and was performed in triplicate. To discriminate against cell debris, dead cells, and living cells, gates of 7.20 μm/13.95 μm (SW1353) and 7.20 μm/14.85 μm (CAL-78) were used.

### 4.4. Proliferation Assay after Indirect CAP-Exposure

In order to have an approximately similar number of cells at the time of the measurement, we pre-incubated an adjusted number of cells for the indirect CAP treatment relative to those in the experiments with direct treatment. Thus, 5 × 10^3^ (SW1353) and 1 × 10^4^ (CAL-78) cells were each separately pre-incubated over 24 h in a humidified atmosphere at 5% CO_2_ and 37 °C. The culture media was removed carefully, and cells were treated with 200 µL CAP-activated media. For this purpose, 200 µL media were treated with 10, 30, and 60 s CAP or carrier gas argon in a second 24-well plate. Cells counts were performed 4, 24, 48, 72, 96, and 120 h after the indirect CAP-exposure, as described in proliferation assay after direct CAP treatment.

### 4.5. FDA-Uptake Assay

**Figure 7:**
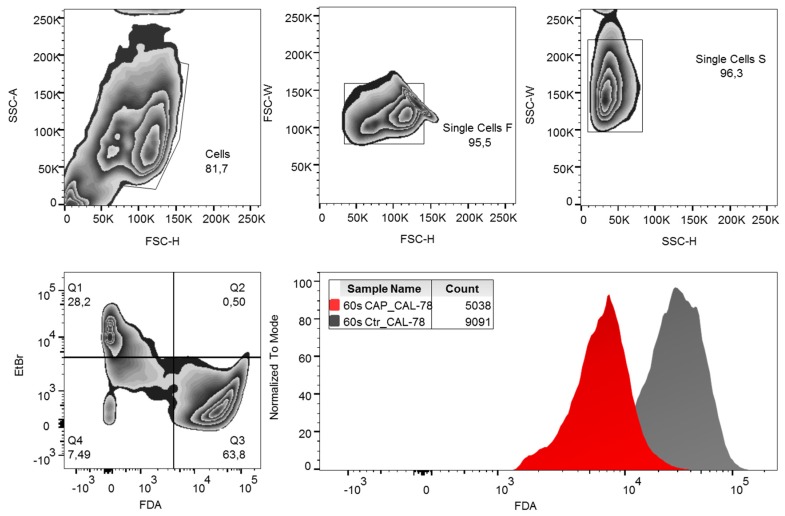
Gating strategy. Fluorescein diacetate (FDA) content per cell was analyzed by flow cytometry. Debris and doublets were excluded by forwarding and side-scatter characteristics. Living cells were defined as ethidium bromide (EtBr)-negative and FDA-positive events. For analysis of data, the mean fluorescence intensity (MFI) of FDA was compared. SSC-A: side-scatter area, SSC-H: side-scatter height, FSC-W: forward-scatter width, FSC-H: forward-scatter height.

Cells were harvested and diluted to 1 × 10^6^ cells per milliliter with stop solution (Dulbecco’s Phosphate-Buffered Saline (DPBS) with 10% FCS *v*/*v*). The cell suspension was stored on ice until use. A dye solution containing 30 μg/mL ethidium bromide and 5 μg/mL FDA in DPBS was used for flow cytometric measurement. The ethidium bromide determination was used to discriminate against dead cells. A total of 200 μL CAP or control cells were added to 200 μL ethidium bromide/FDA dye solution and incubated for 15 min in the dark on ice. After centrifugation (5 min, 300× *g*, 4 °C) the labeled cells were resuspended in DPBS buffer and analyzed in a FACSCanto flow cytometer (BD Biosciences, Heidelberg, Germany) with FACSDiva 6.0 Software (BD Biosciences) and evaluated with FlowJo Software Version 10 (Tree Star Inc., Ashland, OR, USA). The gating strategy used is shown in Figure 7. Normalization of the data of the CAP-treated cells to the control cells was performed.

### 4.6. FDA-Release Assay

An acetone stock solution with 10 mg/mL FDA was prepared for the detection of the bleeding of FDA from FDA-loaded cells and diluted to a concentration of 5 μL/mL with DPBS buffer for the measurement. The cells were loaded with FDA for 30 min in the dark on ice. FDA not incorporated into the cells was removed by washing three times (3 min, 150× *g*, 4 °C) and resuspended in DPBS. A total of 200 μL of cell suspension was treated with CAP or carrier gas argon for 60 s and incubated on ice for 20 min in the dark. Subsequently, the cells were sedimented (3 min, 150× *g*, 4 °C) and 100 μL of the cell-free supernatant was analyzed in an Infinite M200 PRO multimode reader (Tecan, Männedorf, Switzerland) with i-control 1.9 software (Tecan) with an excitation wavelength of 300 nm and an emission wavelength of 520 nm. The CAP treatment measurements were normalized to the control measurements.

### 4.7. ATP-Release Assay

Cells were harvested and adjusted to 10^6^ cells/ml with DPBS 200 μL cell suspension were treated with CAP or carrier gas argon (control group) for 15, 30, and 60 s. After treatment, the cells were incubated over 3 min and sedimented (5 min, 300× *g*, 4 °C). Relative ATP concentrations of the cell-free supernatants were measured using the CellTiter-Glo 2.0 Reagent (Promega GmbH, Walldorf, Germany) and Infinite M200 reader (Tecan, Männedorf, Switzerland). The CAP treatment measurements were normalized to the control measurements.

### 4.8. Dextran-Uptake Assay

Cells were seeded on cover slides and incubated over 24 h (37 °C, 5% CO_2_, humidified atmosphere). After incubation, cells were washed in DPBS and treated with 15, 30, and 60 s CAP or carrier gas argon. Cells were dyed with a staining solution (150 µg/mL FITC-dextran; Sigma-Aldrich, St. Louis, Missouri, USA) and 0.5 µg/mL DAPI (4′,6-Diamidino-2-Phenylindole, Dihydrochloride, Thermo Fisher Scientific, Waltham, MA, USA) in DPBS. After staining, the cells were washed three times with DPBS. Fluorescence of FITC and DAPI were recorded with BZ-9000 microscope and analyzed with a BZ-II Analyzer (KEYENCE, Neu-Isenburg, Germany). The FITC-positive areas were measured and normalized to the count of DAPI-labeled cell cores.

### 4.9. G-/F-Actin Assay

Cells were seeded on cover slides and incubated over 24 h (37 °C, 5% CO_2_, humidified atmosphere). The culture media was changed, and cells were treated with 5 (CAL-78) and 10 s (SW1353) CAP or carrier gas argon. After an incubation period of 4 h, the cells were washed with DPBS and fixed with 1% paraformaldehyde (Carl Roth, Karlsruhe, Germany). Cells were permeabilized with Triton-X100 (0.3%) Carl Roth, Karlsruhe, Germany). Cells were dyed over 20 min with rhodamine-conjugated phalloidin (0.022 µM; Thermo Fisher Scientific, Waltham, MA, USA) and Alexa Fluor 488-conjugated deoxyribonuclease I (0.3 µM; Thermo Fisher Scientific, Waltham, MA, USA) in the dark. DAPI (1.43 µM; Thermo Fisher Scientific, Waltham, MA, USA) was added for the last 3 min of incubation. Fluorescence was recorded with a BZ-9000 microscope and analyzed with a BZ-II Analyzer (KEYENCE, Neu-Isenburg, Germany). The ratio of the red signal compared to the green signal of each cell was calculated.

## 5. Conclusions

The present study, for the first time, demonstrates the effects of plasma treatment in chondrosarcoma cells in vitro. This method could represent an enhancement of the therapeutic spectrum. Significant inhibition of the cell growth of the chondrosarcoma cells was evident even after brief CAP treatment. In addition, damage to the physiology and integrity of the cell membrane and the cytoskeleton of the CS cells was demonstrated. The antiproliferative effect of CAP treatment in combination with the well-known wound healing promoting and antiseptic effects of CAP may represent a promising therapeutic supplement in oncological surgery.
